# *RcPAL,* a key gene in lignin biosynthesis in *Ricinus communis* L.

**DOI:** 10.1186/s12870-019-1777-z

**Published:** 2019-05-06

**Authors:** Jiannong Lu, Yuzhen Shi, Weijin Li, Sen Chen, Yafei Wang, Xiaolin He, Xuegui Yin

**Affiliations:** 1 College of agricultural sciences, Guangdong ocean university, Zhanjiang, 524088 China; 2 College of Chemistry and Environment, Guangdong Ocean University, Zhanjiang, 524088 China; 3 College of Life Science and Technology, Lingnan Normal University, Zhanjiang, 524048 China

**Keywords:** *Ricinus communis* L., *RcPAL*, Amplification, Transformation, Overexpression, Antisense expression, Lignin

## Abstract

**Background:**

Castor (*Ricinus communis* L.) is an important seed oil crop. Castor oil is a highly demanded oil for several industrial uses. Current castor bean varieties suffer from low productivity and high risk of insect pests and diseases. High productive and pest/disease resistance varieties are needed. Lignin has been associated to the resistance for pest, disease and lodging. Lignin is produced from several metabolites of the phenylpropanoid pathway. PAL is the key enzyme of the phenylpropanoid pathway. The gene *PAL* may assist in the improvement of resistance of castor bean.

**Results:**

The *RcPAL* CDs was amplified and its function was examined by transgenic overexpression and antisense expression, lignin histochemical staining, real-time PCR, lignin content measurement and morphological investigation. Its full length was 2145 bp, encoding 714 amino acids. The overexpression of *RcPAL* (7.2 times) increased significantly the PAL activity, dyeing depth of xylem cells and lignin content (14.44%), resulting in a significantly lower plant height, deeper and thicker blade, more green leaves, shorter internode, thicker stem diameter, and opposite in antisense expression plants (lignin content lowered by 27.1%), demonstrated that the gene *RcPAL* was a key gene in castor lignin biosynthesis.

**Conclusions:**

The gene *RcPAL* is a key gene in castor lignin biosynthesis and can be induced to express under mechanical damage stress. When up-regulated, it increased the lignin content significantly and dwarfed the plant height, and opposite when down-regulated. The gene *RcPAL* may assist in the improvement of resistance and plant type of castor bean.

## Background

Castor (*Ricinus communis* L.), belonging to *Ricinus* family, Euphorbiaceae, is an important industrial oil crops in the world. Due to its unique chemical properties, castor oil is widely used in aviation, aerospace, machinery manufacturing, pharmaceutical, chemical and other industries with more than 700 industrial uses [1]. The demand for castor oil in the world is rising at 3~5% per annum [1], but current castor varieties suffer from low productivity and high risk of insect pests and diseases [2, 3]. Castor bean is prone to lodging due to its tall plant type and hollow stem [4]. High productive varieties with resistance to pest, disease and lodging are needed.

Lignin has been associated to pest/disease resistance [5–7] and lodging resistance [8–10]. The resistant varieties generally have the characters such as stem tenacity, wax layer, thick leaf, deep leaf color and developed sclerenchyma, most of which are related to cell wall development and the accumulation of lignin [11, 12]. Lignin is produced from several metabolites of the phenylpropanoid pathway [13, 14]. Phenylalanine ammonia-lyase (PAL) is the key enzyme of the phenylpropanoid pathway [5, 15–17]. Under stress of exogenous signal compounds, mechanical damage, bacteria, viruses, pests, etc., the expression of gene *PAL* can be induced at the transcriptional level and the PAL activity will be increased rapidly to activate phenylpropanoid metabolism in defense system [18–22].

Most of the reported studies has focused on phenylalanine ammonia-lyase, however, limited work was done on the expression of the gene *PAL* itself. Transferring the soybean gene *PAL* into tobacco resulted in the down regulation of gene *PAL* expression, along with stunted growth, scab, abnormality in leaf shape and flower development, decreased pollen fertility [23]. Reducing expression of gene *PAL* by antisense oligonucleotide technique delayed the growth of *Medicago sativa* L. [24]. The total flavonoids content was increased obviously after transferring the gene *PAL* cloned from particularly high content rice into low content rice [25]. In this study, the full length cDNA of gene *PAL* was obtained by RT-PCR method from HY1, a special castor resistant accession, the overexpression and antisense plant expression vector were constructed and the transgenic plants were obtained by acupuncture-vacuum infiltration assisted *Agrobacterium tumefaciens* mediated method to analyze the expression of gene *PAL* and the relationship between the expression of gene *PAL* and accumulation of lignin in castor.

## Results and discussion

### Amplification of *RcPAL* CDs

**Fig. 1:**
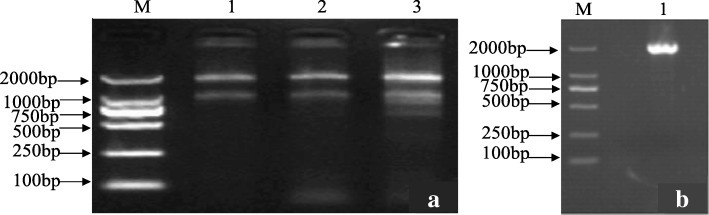
Electrophoresis profile of total RNA and amplified *RcPAL* CDs. **a** Electrophoresis profile of total RNA. M: DL2000 DNA Marker; Lane 1, 2, 3: RNA samples from leaves. **b** Amplification of *RcPAL*A CDs. M: Marker DL2000; Lane 1: PCR product by primers *RcPAL*-F-1/*RcPAL*-R-1

The extracted total RNA was detected with 1% agarose gel electrophoresis and no degradation was found (Fig. 1a), with OD_260_/OD_280_ ratios between 1.8~2.0 and OD_260_/OD_230_ ratios over 2, indicating that the purity and integrity of extracted RNA satisfied the experimental requirements. A specific band of ~ 2150 bp was amplified from cDNA template (Fig. 1b), consistent with the size of *RcPAL* CDs released by NCBI, with a homology of 99, 92 and 91% with *Ricinus communis* (XM_002519475.1), *Jatropha curcas* (DQ883805.1) and *Manihot esculenta* (AF383152.1) respectively (Table 1). It also had a homology of 87% with the gene *PAL*1 (HQ331118), one of the three *PAL* genes in *Epimedium brevicornu* Maxim., which was involved in lignin synthesis [26].

**Table 1:** *PAL* gene CDs nucleotide sequence blastn

Description	Max score	Total score	Query cover	E value	Ident	Accession
Phenylalanine ammonia-lyase [*Ricinus communis*]	1482	1482	99%	0.0	100%	AGY49231.1
Phenylalanine ammonia-lyase [*Ricinus communis*]	1476	1476	99%	0.0	99%	XP002519521.1
Phenylalanine ammonia-lyase [*Jatropha curcas*]	1368	1368	99%	0.0	92%	XP012082374.1
Phenylalanine ammonia-lyase [*Manihot esculenta*]	1358	1358	99%	0.0	92%	XP021660472.1

### Construction of expression vectors

**Fig. 2:**
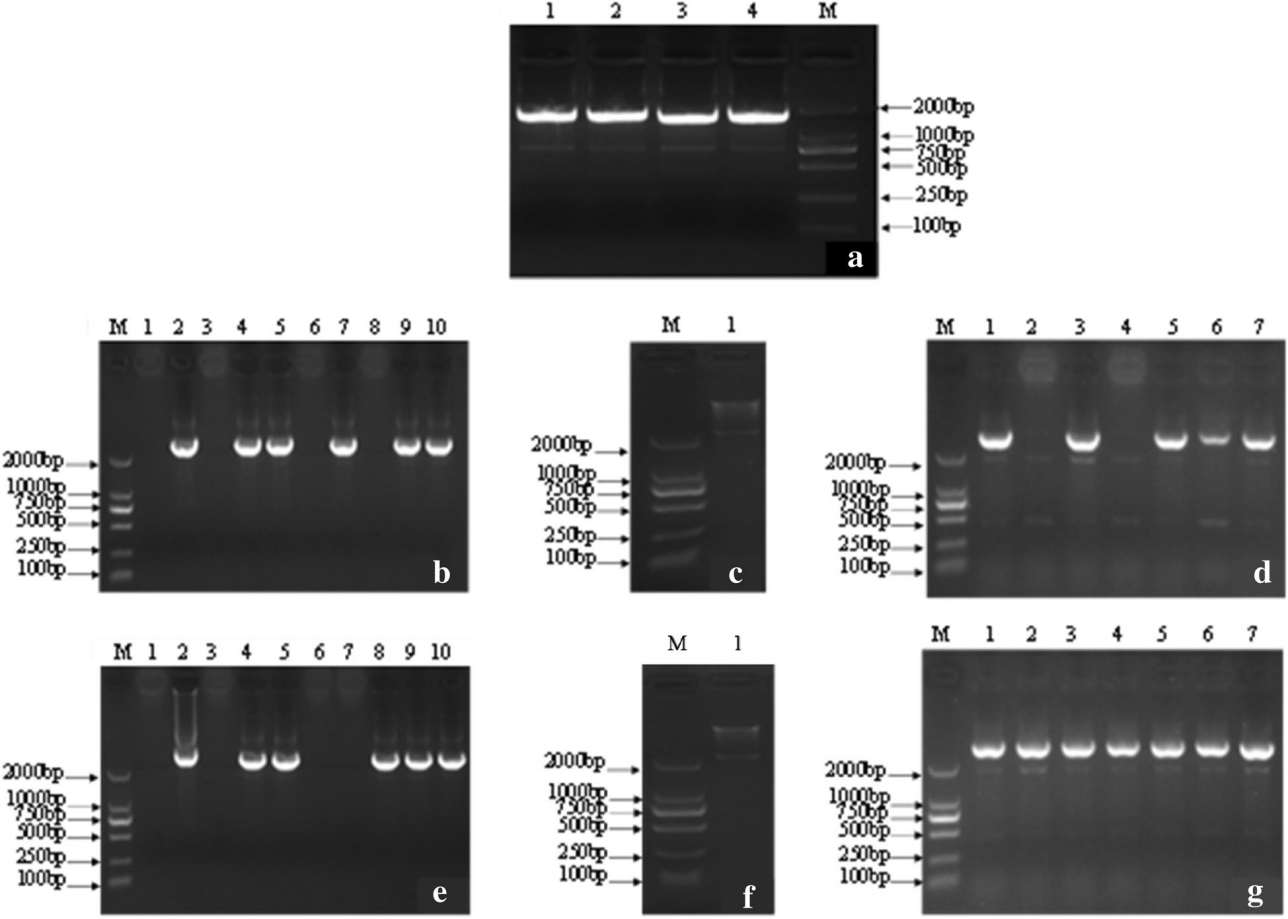
Amplification profiles of *RcPAL* CDs, digested vectors and transformed EHA105. **a** Amplification of *RcPAL* CDs. Lane M: Marker DL2000; lanes 1~2: overexpression CDs; lanes 3~4: antisense CDs. **b** Amplification of pYLRNAi-*PAL*^+^. Lane M: Marker DL2000; lane 1: negative control pYLRNAi.02; lane 2: positive control pMD-*PAL*; lanes 3~10: recombinant vectors, among which 4, 5, 7, 9, 10 were positive. **c** Double digestion of pYLRNAi-*PAL*^*+*^. Lane M: Marker DL2000; lane 1: double digestion products. **d** Amplification of *Agrobacterium* EHA105 with pYLRNAi-*PAL*^*+*^. Lane M: Marker DL2000; Lane 1: positive control pYLRNAi-PAL^+^; lane 2~7: transformed *Agrobacterium* EHA105, among which 3, 5, 6 and 7 were positive. **e** Amplification of pYLRNAi-*PAL*^−^. Lane M: Marker DL2000; lane 1: negative control pYLRNAi.02; lane 2: positive control pMD-*PAL*; lanes 3~10: recombinant vectors, among which 4, 5, 8, 9, 10 were positive. **f** Double digestion of pYLRNAi-*PAL*^*−*^. Lane M: Marker DL2000; lane 1: double digestion products. **g** Amplification of *Agrobacterium* EHA105 with pYLRNAi-*PAL*^−^. Lane M: Marker DL2000; lane 1: positive control pYLRNAi-PAL^−^; lane 2~7: transformed *Agrobacterium* EHA105, all were positive

The overexpression vector pYLRNAi-*PAL*^+^ and antisense expression vector pYLRNAi-*PAL*^−^ were constructed. Firstly, the *RcPAL* CDs were amplified from the cDNA template, the PCR products had the same size as the expected maximum *RcPAL* ORF (open read frame) (Fig. 2a). Secondly, a band of 2.2 kb was amplified from the the cloning vectors of pMD-*PAL* (Fig. 2b, Fig. 2e), proving that the coding sequence has been inserted the vector. Thirdly, the band of 2.2 kb was also obtained from the digested products of recombinant vectors pYLRNAi-*PAL*^+^ and pYLRNAi-*PAL*^−^ (Fig. 2c, Fig. 2f), which were constructed by double digesting the pMD-*PAL* and pYLRNAi2.0 by *Bgl*IIand *Mlu* I and connecting the target fragments with pYLRNAi 2.0, demonstrating that the expression vectors has been constructed successfully. Finally, a bright band of 2.2 kb was amplified from the transformed *Agrobacterium tumefaciens* EHA105 (Fig. 2d, Fig. 2g), proving the successful transformation of pYLRNAi-*PAL*^+^ and pYLRNAi-*PAL*^−^.

### Identification of transgenic plants

**Fig. 3:**
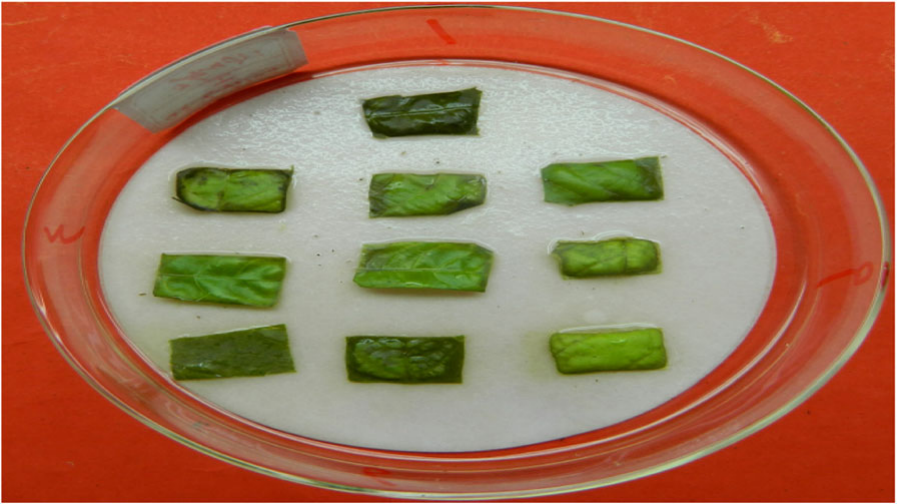
Hygromycin screening of transgenic plants

**Fig. 4:**
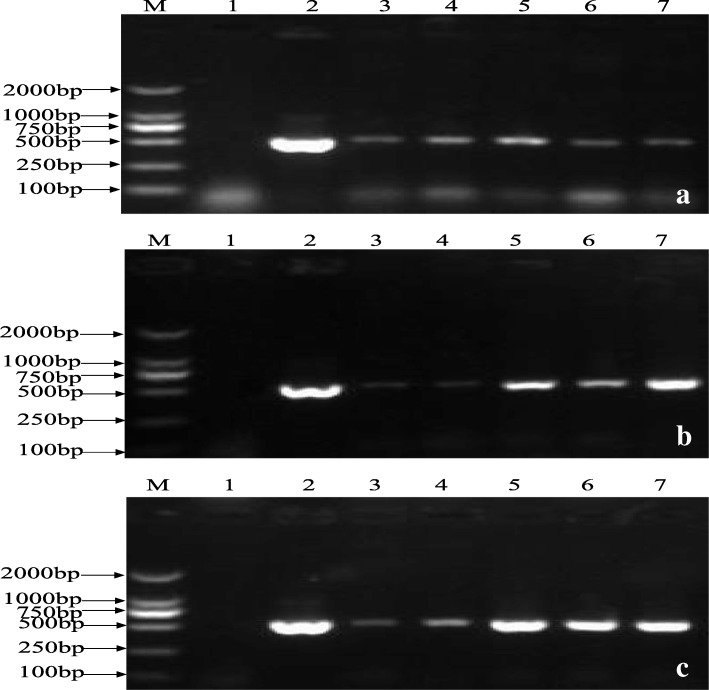
PCR identification of of transgenic plants with sense, antisense and empty vector. **a** pYLRNAi.02; **b** pYLRNAi-*PAL*^+^; **c** pYLRNAi-*PAL*^−^. Lane M: Marker DL2000; Lane 1: negative control (untransformed); Lane 2: positive control (pYLRNAi.02); Lanes 3~7: positive transformed plants

Overall, 79, 85 and 93 positive plants transformed by pYLRNAi-*PAL*^+^, pYLRNAi-*PAL*^−^ and pYLRNAi.02 respectively were screened out by both hygromycin detection (Fig. 3) and PCR identification (*Hyg*-F/*Hyg*-R) in which a target band of 520 bp was amplified (Fig. 4), with a transformation rate of 82.3, 81.0 and 85.3% respectively, which showed that the exogenous DNA has been integrated into the castor genome.

### Histochemical staining of lignin in transgenic plants

**Fig. 5:**
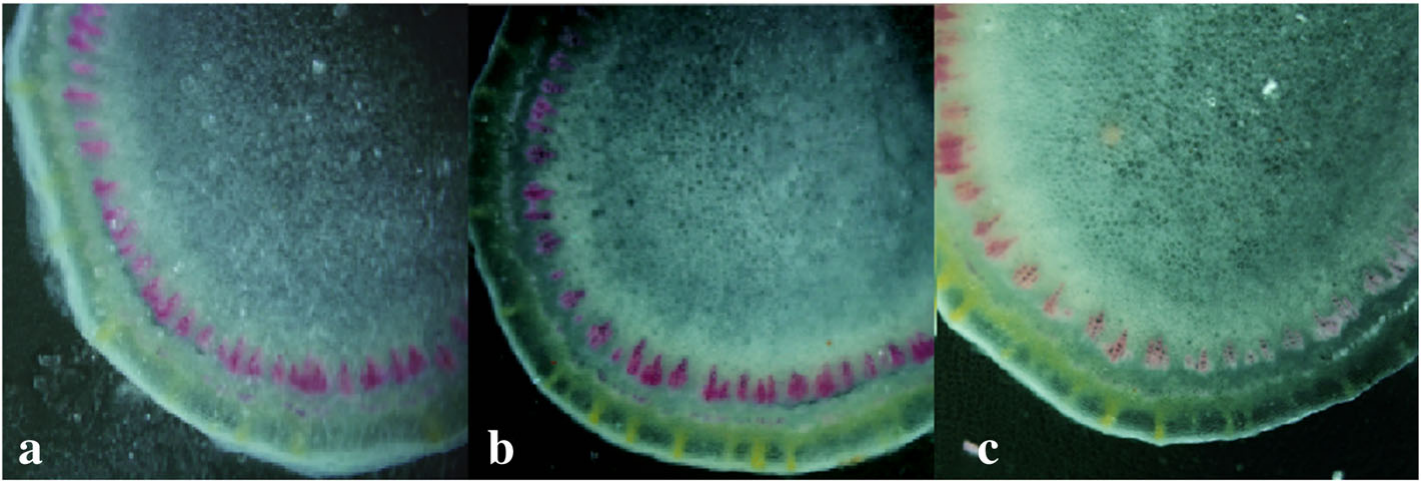
Wiesner staining of transformant petiole. **a** WTs (35s^0^–5) × 400; **b** OPs (35S^+^-18) × 400; **c** APs (35 s^−^-9) × 400

**Fig. 6:**
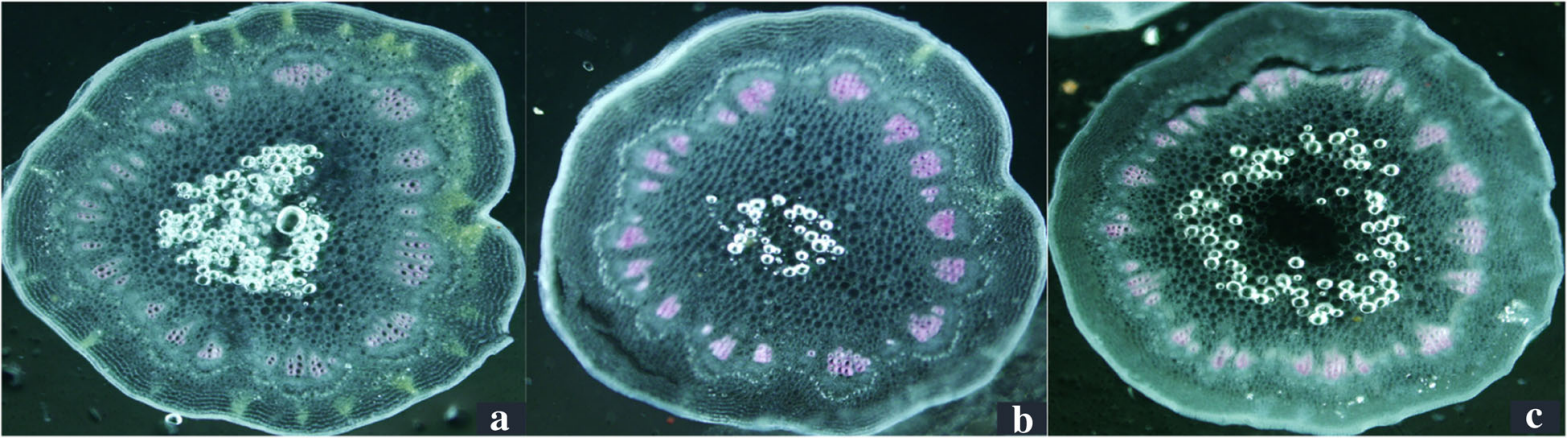
Wiesner staining of transformant stalk top. **a** WTs (35s^0^–5) × 400; **b** OPs (35S^+^-18) × 400; **c** APs (35 s^−^-9) × 400

The xylem cell of petiole and stalk top in overexpression plants (OPs) (35S^+^-18) were stained deeper than wild type plants (WTs) (35s^0^–5) and antisense expression plants (APs) (35 s^−^-9), just WTs were a little deeper than APs (Fig. 5, Fig. 6). The dyeing depth reflected the lignin content in tissue, which was consistent with the expression of gene *RcPAL*.

### Morphological differences between OPs, APs and WTs

**Fig. 7:**
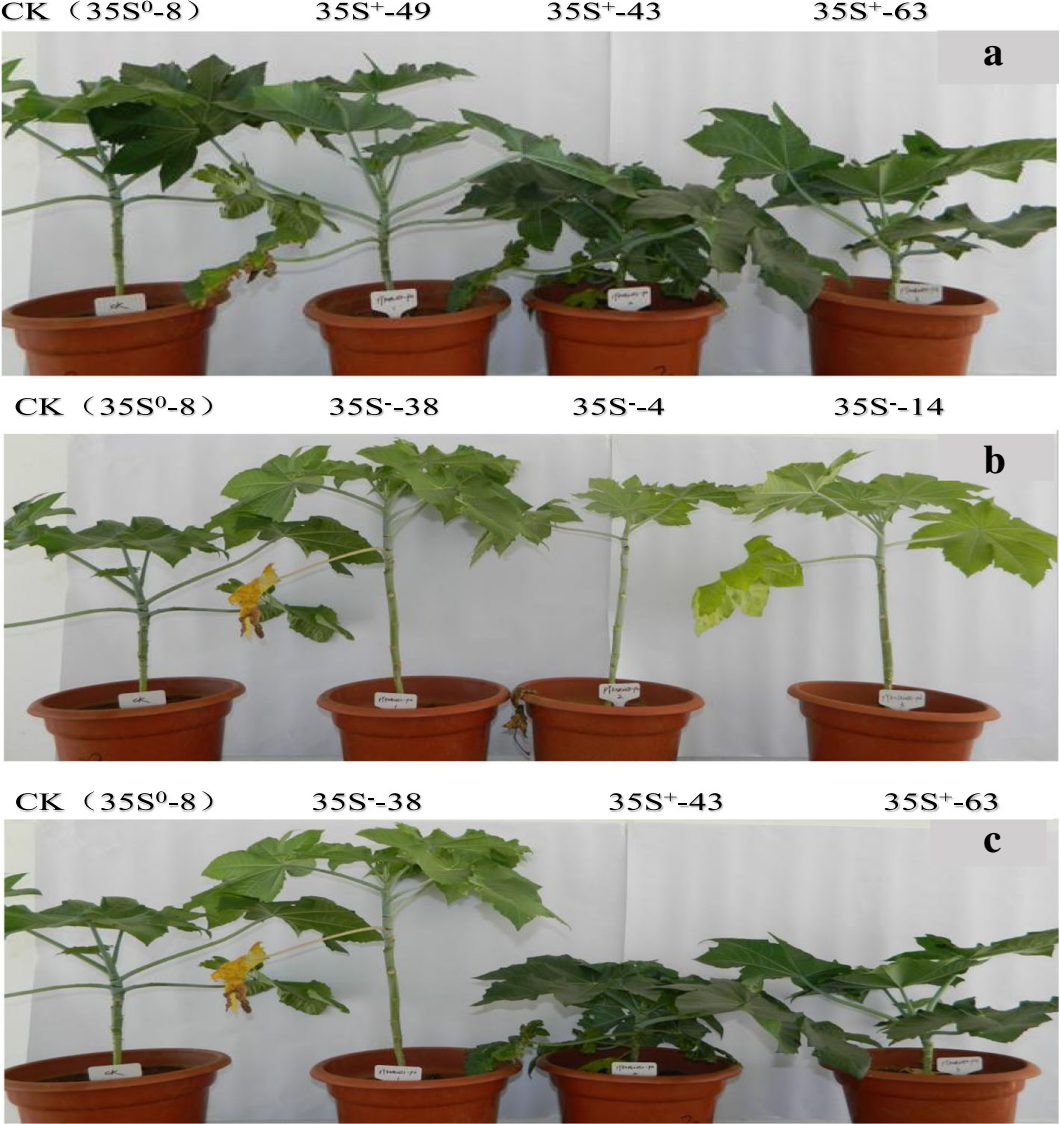
Morphological characteristics of different transformants. **a** Control and OPs; **b** Control and APs; **c** Control, OP and APs

As showed in Fig. 7, compared with WP (35S^0^–8), the OPs (35S^+^-49, 35S^+^-43, 35S^+^-63) was significantly changed on plant type, exhibited a lower plant height, deeper and thicker blade, more green leaves, shorter internode, thicker stem diameter and extended mature period, but opposite in APs (35S^−^-38, 35S^−^-4, 35S^−^-14) except for mature period (Table 2).

**Table 2:** Comparison of morphological characteristics between different transformants

Individual	Plant height (mm)		Main stem height (mm)		Stem diameter (mm)		Node number		Internode length (mm)		Green leaf number		Leaf thickness (mm)		Mature period (d)	
	Value	%	Value	%	Value	%	Value	%	Value	%	Value	%	Value	%	Value	%
35S^0^–8	660	–	415	–	17.54	–	25	–	16.6	–	7	–	0.62	–	155	–
35S^+^-43	370	−43.94	240	−41.17	18.5	5.47	25	0	9.6	−42.17	9	28.57	0.88	41.94	187	20.65
35S^+^-49	540	−18.18	240	−41.17	18.23	3.93	25	0	9.6	−42.17	7	0.00	0.72	16.13	187	20.65
35S^+^-63	520	−21.21	310	−25.30	18.24	3.99	25	0	12.4	−25.30	9	28.57	0.71	14.52	187	20.65
35S^−^-4	830	25.76	570	37.35	17.82	1.60	25	0	22.8	37.35	6	−14.29	0.6	−3.23	155	0.00
35S^−^-14	860	30.3	510	22.89	16.3	−7.07	25	0	20.4	22.89	6	−14.29	0.56	−9.68	155	0.00
35S^−^-38	1000	51.52	710	71.08	14.7	−16.19	25	0	28.4	71.08	6	−14.29	0.51	−17.74	155	0.00

Up-regulation or down-regulation of *RcPAL* expression can accelerate or inhibit the production of intermediate products in phenylpropane pathway such as trans-cinnamic acid, coumaric acid, ferulic acid, erucic acid and so on [27]. These products can be transformed into coumarin and chlorogenic acid or trans-coumarin-CoA ester which will be further transformed into lignin, flavonoids and other substances through several ways to increase or decrease the content of lignin and flavonoids, and ultimately influence the phenotype of transformants [28, 29]. The insertion of overexpression and antisense genes changed the content of lignin and endogenous auxin [30], which lead to a series of phenotypic variation finally. On one hand, the increase of lignin content made OPs possessed darker and thicker leaves and greener leaves; on the other hand, the polar transport inhibition of endogenous auxin and its accumulation in growing point caused the multiple variations on OPs such as reduced plant height, shortened internode, thickened stem and extended maturation period. Because the molecular structure of flavonoids is very similar to that of auxin polar transport inhibitors, it had been considered as endogenous auxin polar transport inhibitors for a long time [30]. The significant difference between OPs probably resulted from the different copy number or insertion site. As for APs, the decrease of lignin content and flavonoid synthesis caused the opposite variations such as higher plant height, lighter and thinner leaf, less green leaves, longer internode and thinner stem, no maturation period changed.

As it can dwarf the plant height by means of shorter main stem height and internode length (Table 2), OPs are expected to facilitate the plant type breeding in castor.

### Determination of PAL activity

**Fig. 8:**
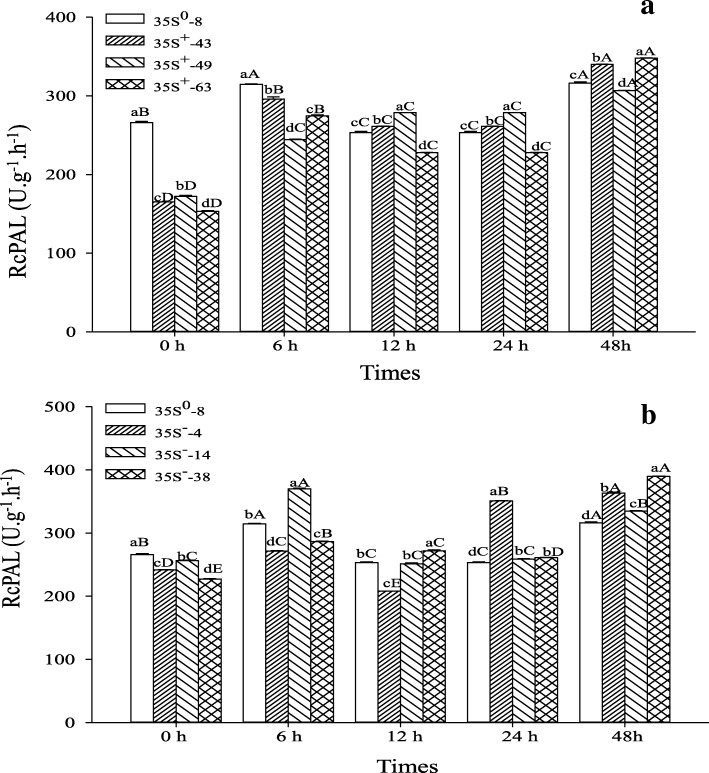
Effect of stab on PAL activity in transformants. **a** OPs; **b** APs. Capital letters indicate the significance (*P* < 0.05) between different times in the same plant; Lower-case letters indicate the significance (P < 0.05) between different plants at the same time

Mechanical damage can result in the increase of PAL activity in plant, it is the response of plant to external stress, which was presumed that the damage signal activated the expression of the defense enzyme genes which furtherly induced the PAL synthesis [30–32]. In this study, the PAL activity in OPs and APs was significantly lower than control before stab treatment (Fig. 8). After stab treatment, it increased firstly, then decreased slightly, and finally reached a peak at 6~48 h, whether in OPs or APs or control. The OPs and APs could respond to mechanical damage stress more rapidly by increasing the PAL activity, the increase was 42.06%~ 79.38 and 12.34%~ 44.41% respectively at 6 h, and 78.18%~ 127.16 and 30.65%~ 71.50% at 48 h respectively, much more than in control. In addition, the increase in OPs was much greater than in APs.

The *RcPAL* can express both constitutively and inductively. Its overexpression or silence can reduce the PAL background activity but can raise it under mechanical damage stress, especially in the case of over expression. The increase was much greater in OPs than in APs and WT, which coincided with the expected results.

### Expression of gene *PAL* under mechanical damage

**Fig. 9:**
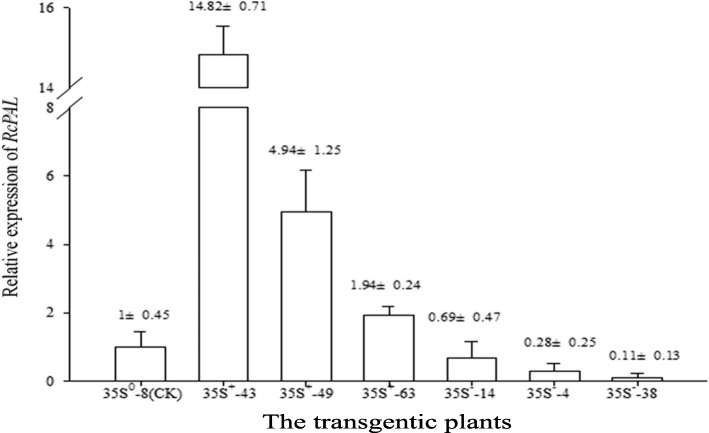
Relative expression of *RcPAL* in different transformants

The *RcPAL* expression significantly increased (up to 14.82 times) in OPs and decreased (down to 0.11 times) in APs in comparison with WT (Fig. 9). There was also significant difference between OPs, which was presumed due to the different sites or copy numbers of inserted gene, but no between APs. As expected, OPs up-regulated the expression of gene *RcPAL*, while opposite in APs.

### Lignin content in transgenic plants

**Fig. 10:**
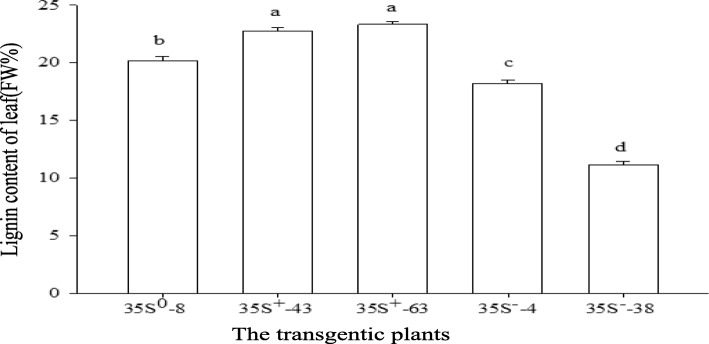
Leaf lignin content in OPs, APs and WTs. Lower-case letters indicate the extreme significance (*P* < 0.01) between different plants

The leaf lignin content was extremely significantly increased in OPs (13.05 and 15.83%) and extremely significantly decreased (9.53 and 44.67%) in APs in comparison with WT (Fig. 10). The overexpression and silencing of gene *RcPAL* resulted in the increase and decrease of lignin content respectively which supported the conclusion that the gene *RcPAL* played a key role in lignin biosynthesis in castor.

## Conclusions

The *RcPAL* is a key gene in castor lignin biosynthesis and can be induced to express under mechanical damage stress. When up-regulated, it can increase the lignin content significantly and dwarf the plant height, and opposite when down-regulated. The *RcPAL* gene may assist the plant breeding for resistance and architecture in castor bean.

## Methods

### Plant and bacterial material

**Fig. 11:**
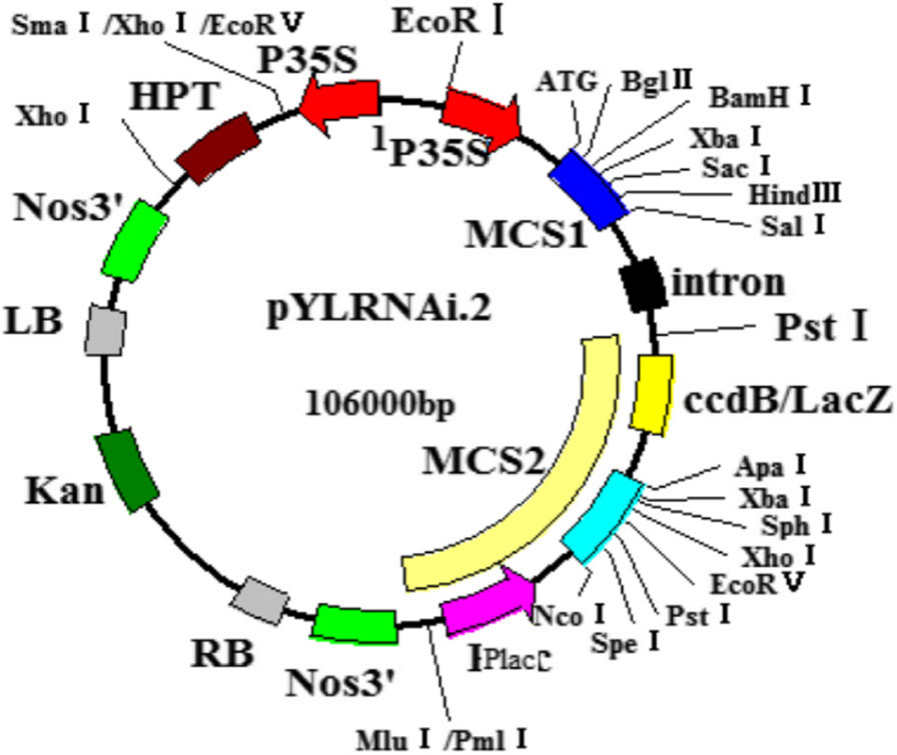
The structure of plant expression vector pYLRNAi.02

The castor material was an inbred line HY1, developed by the laboratory of molecular breeding for energy crops in Guangdong Ocean University. The plant expression vector pYLRNAi.02 (Fig. 11) was provided by Prof. Liu Yaoguang of South China Agricultural University. *Escherichia coli* TOP10 and *Agrobacterium* EHA105 were provided by Prof. Jie Xinming of South China Agricultural University. 2 × *Taq* Master Mix, DNA Marker, LA *Taq*, Ex *Taq*, 10 × Loading buffer, reverse transcription kits were all bought from TaKaRa.

### DNA, RNA extration and cDNA synthesis

Genomic DNA of transgenic plants was extracted with modified CTAB method. The RNA of transgenic receptor was extracted by Trizol method with extract RNAiso Plus and adjuvant RANiso-mate for Plant Tissue (TaKaRa) according to the manual of TRIzol kit. After testing for purity and integrity, genomic DNA residue in RNA was eliminated with DNase I to guarantee RNA purity. The first strand of cDNA was synthesized in accordance with the reverse transcription kit instructions.

### Primer design

Upstream and downstream primers were designed at the start and stop codon regions of *RcPAL* according to the ORF sequence released in GenBank (XM_002519475.1) to amplify *RcPAL* CDs, named *RcPAL*-F-1 and *RcPAL*-R-1. The *Actin* was used as reference gene and its forward and reverse primers were designed, named as *RcACTINs* and *RcACTINa.* The forward and reverse primers of target gene were also designed according to primer design principle of real-time PCR, named *RcPAL-*F-2 and *RcPAL-*R-2*.* A pair of specific primers were designed on hygromycin gene sequence to detect transgenic plants, named *Hyg*-F and *Hyg*-R. The forward and reverse primers for overexpression vector/antisense expression vector (pYLRNAi-*PAL*^+^/ pYLRNAi-*PAL*^−^, after the same) were designed at the start and stop codon regions of *RcPAL* CDs, adding the corresponding restriction site, removing the stop codon (TTA) from the reverse primer for antisense expression vector, named as *RcPAL*^+^-F/*RcPAL*^+^-R and *RcPAL*^−^-F/*RcPAL*^−^-R respectively. Primer sequences were as follows.

*RcPAL-F-1*: 5′-ATGGCAGCAATGGCAGAAAATGGC-3′.

*RcPAL-R-1*: 5′-TTAGCAAATTGGAAGAGGGGC-3′.

*RcACTINs*: 5′-CCCAGCACACAGCAGCAA-3′.

*RcACTINa*: 5′-AGGACTTGAAGAGGAAGAGAGAAACC-3′.

*RcPAL-F-2*: 5′-ATCTGAGGCATCTGGAGGAA-3′.

*RcPAL-R-2*: 5′-CAGCATAGGCAAAGACATACTC-3′.

*Hyg-F*: 5′-GGCGAAGAATCTCGTGCTTTCA-3′.

*Hyg-R*: 5′-CAGGACATTGTTGGAGCCGAAA-3′.

*RcPAL*^*+*^*-F* (Adding *Bgl*IIsite): 5′-GAAGATCTATGGCAGCAATGGCAGAAAATGGC-3′ *RcPAL*^*+*^*-R* (Adding *Mlu*I site): 5′-CGACGCGTTTAGCAAATTGGAAGAGGGGC-3′.

*RcPAL*^*−*^*-F* (Adding *Mlu*I site): 5′-CGACGCGTATGGCAGCAATGGCAGAAAATGGC-3′.

*RcPAL*^*−*^*-R* (Adding *Bgl*IIsite): 5′-GAAGATCTGCAAATTGGAAGAGGGGC-3′.

### Target fragment amplification

Maximum ORF sequence of *RcPAL* was amplified with primers *RcPAL*-F-1 and *RcPAL*-R-1 using HY1 cDNA as template. The PCR reaction procedure was 95 °C 5 min for 1 cycle, 94 °C 35 s, 62 °C 35 s and 72 °C 3 min for 35 cycles; extension for 10 min at 72 °C. PCR products were checked with 1% agarose gel electrophoresis.

### Construction of overexpression and antisense expression vector of *RcPAL*

The overexpression vector pYLRNAi-*PAL*^+^ and antisense expression vector pYLRNAi-*PAL*^−^ were constructed by digesting plant expression vector pYLRNAi.02 (with bacterial screening marker *Kan*^*r*^, and plant screening marker *Hyg*^*r*^) and the target CDs sequences of *RcPAL* with *Bgl*II and *Mlu*I, recycling vector and target fragments and ligating them with T4 ligase. The CDs sequences of *RcPAL* was amplified from pMD-*RcPAL* with the upstream and downstream primers for overexpression and antisense expression respectively. The recombinant vectors were used to transform *Escherichia coli* TOP10.

### Transformation

**Fig. 12:**
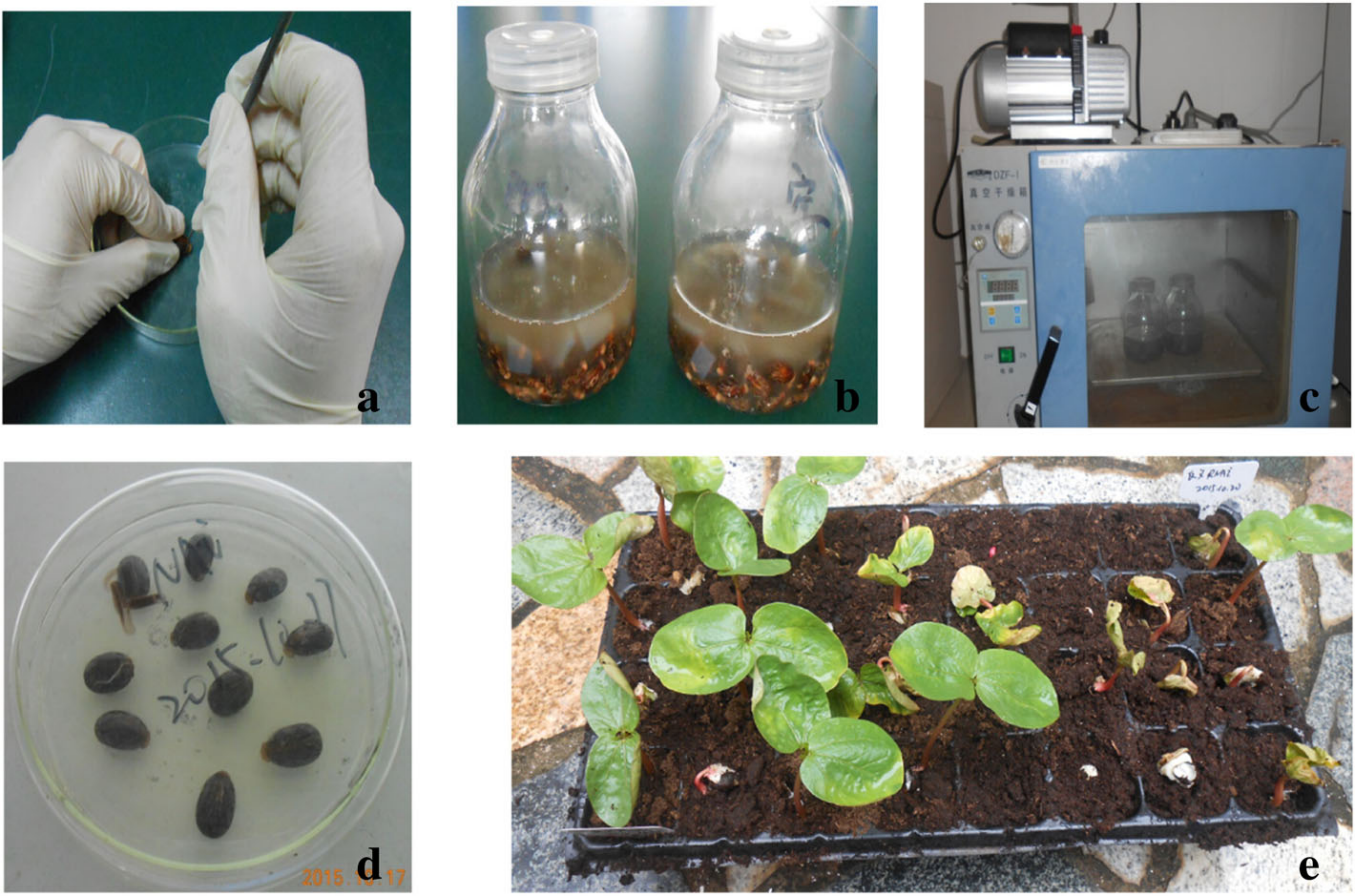
Genetic transformation of castor. **a** Acupuncture; **b** Infection; **c** Vacuum infiltration; **d** Co-cultivation; e: Transfer

Imbibing seeds were transformed with vectors pYLRNAi.02 (wild type), pYLRNAi-*PAL*^+^ and pYLRNAi-*PAL*^−^ respectively by acupuncture-vacuum infiltration assisted *Agrobacterium* tumefaciens mediated method. Firstly, the dry plump castor seeds were sterilized with 70% ethanol for 1 min and then with 10% sodium hypochlorite for 30 min. After being rinsed thoroughly, they were dipped in water of 40 °C for 30 min and transferred onto filter papers previously moistened with distilled water for imbibing at 28 °C for 24 h. Secondly, the seed coat was cracked with an anatomical needle and the seed was pierced once with a disposable syringe of 1 mL(the syringe needle diameter was 0.45 mm)to a depth of ~ 1 mm at the site near the caruncle on the longitudinal midline of seed back, exactly at the the junction of the inclined plane and the plane, beneath which the epicotyl was located. Note that before piercing, the syringe needle had been dipped in the *Agrobacterium* inoculum. In order to inoculate *Agrobacterium* into the embryonic meristem effectively and avoid damaging the growing point, the acupuncture point should be behind the plumule which lay beneath the seed coat where a shoot would emerge later (Fig. 12a). Thirdly, the pierced seeds were then soaked into the *Agrobacterium* inoculum within a air-permeable conical flask (Fig. 12b) and the conical flask was placed into a vacuum compartment, pumped at a pressure of 50 kPa for 20 min, released for 2 min and then vacuum pumped again at same pressure for 5 min (Fig. 12c). Fourthly, the inoculated seeds were transferred without rinsing again onto filter paper moistened with *Agrobacterium* inoculum and further incubated in the dark at 28 °C for 3 days and until beginning of germination after ~ 9 days (Fig. 12d). Finally, the seedlings were immersed into a 250 mg/L carbenicillin solution for 1 h to remove the remnant *Agrobacterium*, and after being rinsed thoroughly with sterile water, they were transplanted to a seedling tray with NOVARBO substrate (Finland)and grown in greenhouse (Fig. 12e). Each vector transformed 150 seeds [33].

### Identification of transgenic plants

#### Hygromycin identification

The third leaf from the top of the transgenic plants with 3~4 leaf was cut into rectangular pieces of 1.5 cm × 1.0 cm and soaked in 16 mg/L hygromycin solution containing 0.5 mg/L 6-BA (screening system established in laboratory). At 4 days later, the individuals with dark stripes or necrotic plaques were negative ones, while those remaining green were primarily judged as positive ones.

#### PCR identification

PCR identification was performed with the primers Hyg*-*F / Hyg-R using the leaf DNA of transgenic plant as template. The PCR reaction procedure was 95 °C 5 min, one cycle; 94 °C 35 s, 55 °C 35S, 72 °C 1 min, 35 cycles and 72 °C 10 min. PCR products were detected by 1% agarose gel electrophoresis.

### Phenotype investigation of transgenic plants

When the transgenic plants were grown in plastic barrels for 80 days, the plant height, stem diameter (base, middle and upper part), stem length, leaf thickness, number of nodes per stem and number of green leaves per plant were investigated.

### Determination of PAL activity in transgenic plants

A total of 3 positive transgenic plants with pYLRNAi-*PAL*^+^, 3 positive transgenic plants with pYLRNAi-*PAL*^−^ and 1 transgenic plant with pYLRNAi.02 were selected at 5-leaf stage for PAL activity analysis. Firstly, each blade of these plants was for 6 times with insect needle 5#. At 0, 6, 12, 24 and 48 h after stabbing respectively, 200 mg of fresh leaf tissue was taken from the third leaf from the top on the main stem of each plant, repeated 3 times. The samples were quickly frozen in liquid nitrogen and stored at − 80 °C. Secondly, each sample was ground into homogenate in an ice-cooled mortar with 1 ml of enzyme extraction buffer (0.05 mol/L boric acid, 5.0 mmol/L β-mercaptoethanol, 1.0 mmol/L EDTA-Na_2_, 5% glycerinum and 5% PVP). The homogenate was transfered into a 2 mL centrifuge tube, setting volume to 2 mL with enzyme extraction buffer, vibrating for 1 min, and centrifuged at 10,000 rpm at 4 °C for 15 min. The supernatant was collected as sample solution for enzyme assay. Thirdly, PAL activity was determined based on the rate of cinnamic acid production as described by Ochoa-Alejo [34]. Briefly, 1 mL 0.02 mol/L L-phenylalanine and 2 mL 0.1 mol/L H_3_BO_3_ buffer were added into a 4 mL centrifugal tube, besides, 0.5 mL sample solution was added into the measuring tube and 0.5 mL enzyme extraction buffer was added into the control tube. After water bathing at 30 °C for 60 min, the reaction was terminated by adding 0.2 mL 6 mol/L HCl. With the control tube adjusting zero, the absorbance A_290_ of the reaction liquid in measuring tube was measured at the wavelength of 290 nm, 1 U of enzyme activity equals to 0.01 of A_290_ value increased per min.

$$ \mathrm{PAL}\ \mathrm{activity}\left[\mathrm{U}/\left( gFw\cdotp h\right)\right]=\frac{A_{290}\times Vt\times V}{0.01\times Vs\times Fw\times t} $$

(*A290*: absorbance; *Vt*: Total volume of the enzyme fluid; Vs: The quantity of the enzyme fluid taken for measurement; *V*: Total volume of reaction liquid; *t*: Reaction time; *Fw*: Fresh weight of sample) [35].

### Histochemical staining of lignin

3 positive transgenetic plants with pYLRNAi.02, pYLRNAi-*PAL*^+^ and pYLRNAi-*PAL*^−^ respectively were selected for histochemical staining of lignin with Wiesner staining method. Wiesner staining method was as follows: prepared freehand tissue slice (50~100 μm) and soaked them in 2% (*v*/v) phloroglucin (dissolved in absolute ethanol) for 5 min, then immersed in 12% (v/v) hydrochloric acid for 5 min, finally, fixed on the slide to be observed and photographed by microscope.

### Determination of gene expression

After 24 h of mechanical stab treatment in transgenic plants, the total RNA of leaves with pYLRNAi.02, pYLRNAi-*PAL*^+^ and pYLRNAi-*PAL*^−^ were extracted respectively. The synthesis of cDNA was performed according to the instructions of PrimeScript® RT reagent Kit with gDNA Eraser (Perfect Real Time) (Takara) with the castor gene *Actin* as internal reference. Quantitative PCR program was 95°Cfor 30s (20 °C/s) of 1 cycle; 95°C for 5 s (20 °C/s), 60 °C for 30s (20 °C/s) of 40 cycles; 95 °C for 0 s (20 °C/s), 60 °C for 15 s (20 °C/s), 95 °C for 0 s (0.1 °C/s) of 1 cycle. The melting curve was checked after completion and relative expression was calculated with 2^-△△CT^ method.

### Determination of total lignin

The determination of total lignin content was carried out in accordance with acetyl bromide method [35].
